# Ultrashort electromagnetic pulse control of intersubband quantum well transitions

**DOI:** 10.1186/1556-276X-7-478

**Published:** 2012-08-23

**Authors:** Emmanuel Paspalakis, John Boviatsis

**Affiliations:** 1 Materials Science Department, School of Natural Sciences, University of Patras, Patras, 26504, Greece; 2 Technological and Educational Institute of Patras, Patras, 26334, Megalou Alexandrou 1, Greece

**Keywords:** Coherent control, Semiconductor quantum well, Intersubband transition, Ultrashort electromagnetic pulse

## Abstract

We study the creation of high-efficiency controlled population transfer in intersubband transitions of semiconductor quantum wells. We give emphasis to the case of interaction of the semiconductor quantum well with electromagnetic pulses with a duration of few cycles and even a single cycle. We numerically solve the effective nonlinear Bloch equations for a specific double GaAs/AlGaAs quantum well structure, taking into account the ultrashort nature of the applied field, and show that high-efficiency population inversion is possible for specific pulse areas. The dependence of the efficiency of population transfer on the electron sheet density and the carrier envelope phase of the pulse is also explored. For electromagnetic pulses with a duration of several cycles, we find that the change in the electron sheet density leads to a very different response of the population in the two subbands to pulse area. However, for pulses with a duration equal to or shorter than 3 cycles, we show that efficient population transfer between the two subbands is possible, independent of the value of electron sheet density, if the pulse area is *Π*.

## Background

The coherent interaction of electromagnetic fields with intersubband transitions in semiconductor quantum wells has led to the experimental observation of several interesting and potentially useful effects, such as tunneling-induced transparency [1,2], electromagnetically induced transparency [3], Rabi oscillations [4,5], self-induced transparency [5], pulsed-induced quantum interference [6], Autler-Townes splitting [7,8], gain without inversion [9], and Fano signatures in the optical response [10]. In most of these studies, atomic-like multi-level theoretical approaches have been used for the description of the optical properties and the electron dynamics of the intersubband transitions.

Many-body effects arising from the macroscopic carrier density have also been included in a large number of theoretical and experimental studies of intersubband excitation in semiconductor quantum wells [6,10-38]. These studies have shown that the linear and nonlinear optical responses and the electron dynamics of intersubband quantum well transitions can be significantly influenced by changing the electron sheet density.

An interesting problem in this area is the creation of controlled population transfer between two quantum well subbands [23-27,29,30]. This problem was first studied by Batista and Citrin [23] including the many-body effects arising from the macroscopic carrier density of the system. They showed that the inclusion of the electron-electron interactions makes the system behave quite differently from an atomic-like two-level system. To have a successful high-efficiency population transfer in a two-subband, n-type, modulation-doped semiconductor quantum well, they used the interaction with a specific chirped electromagnetic field, i.e., a field with time-dependent frequency. They showed that a combination of *Π* pulses with time-dependent frequency that follow the population inversion can lead to high-efficiency population inversion. Their method was refined in a following publication where only linearly chirped pulses were used for high-efficiency population transfer [27] and was also applied to three-subband quantum well systems [26].

Different approaches for creating high-efficiency intersubband population transfer were also proposed by our group [24,25,29,30]. Using analytical solutions of the effective nonlinear Bloch equations [20], under the rotating wave approximation, we presented closed-form analytical solutions for the electric field amplitude of the electromagnetic field that leads to high-efficiency population transfer [24,25]. In addition, closed-form conditions for high-efficiency transfer were also presented [24,29]. Moreover, efficient population transfer is found when a two-subband system interacts with a strong chirped electromagnetic pulse, for several values of the chirp rate and the electric field amplitude [30].

In this article, we continue our work on the creation of high-efficiency controlled population transfer in intersubband transitions of semiconductor quantum wells. We give emphasis to the case of interaction of the semiconductor quantum well with electromagnetic pulses with a duration of few cycles and even a single cycle. We numerically solve the effective nonlinear Bloch equations [20] for a specific double GaAs/AlGaAs quantum well structure, taking into account the ultrashort nature of the applied field, and show that high-efficiency population inversion is possible for specific pulse areas. The dependence of the efficiency of population transfer on the electron sheet density and the carrier envelope phase of the pulse is also explored. More specifically, we find that for electromagnetic pulses with duration of several cycles, the change in the electron sheet density leads to a very different response of the population in the two subbands to pulse area. However, a *Π* pulse with a duration equal to or shorter than 3 cycles can lead to efficient population transfer between the two subbands independent of the value of electron sheet density.

We note that the interaction of ultrashort electromagnetic pulses with atoms has been studied in the past decade, giving emphasis either to ionization effects [39-41] or to population dynamics in bound two-level and multi-level systems [40,42-46]. Also, the interaction of ultrashort electromagnetic pulses with intersubband transitions of semiconductor quantum wells has been recently studied [47,48], but without taking into account the effects of electron-electron interactions in the system dynamics.

## Methods

The system under study is a symmetric double semiconductor quantum well. We assume that only the two lower energy subbands, *n *= 0 for the lowest subband and *n *= 1 for the excited subband, contribute to the system dynamics. The Fermi level is below the *n *= 1 subband minimum, so the excited subband is initially empty. This is succeeded by a proper choice of the electron sheet density. The two subbands are coupled by a time-dependent electric field *E*(*t*). Olaya-Castro et al. [20] showed that the system dynamics is described by the following effective nonlinear Bloch equations:

(1) $$\begin{array}{ll} {\dot{S}}_{1}(t)\; & =\;[\omega_{10}-\gamma S_{3}(t)]S_{2}(t)-\frac{S_{1}(t)}{T_{2}}\;,\; \end{array}$$

(2) $$\begin{array}{lll} {\dot{S}}_{2}(t)\; & =\;-[\omega_{10}-\gamma S_{3}(t)]S_{1}(t)+2[\frac{\mu E(t)}{\hbar }-\beta S_{1}(t)]S_{3}(t)\; & \;\\ \; & -\;\frac{S_{2}(t)}{T_{2}}\;,\; \end{array}$$

(3) $$\begin{array}{ll} {\dot{S}}_{3}(t)\; & =\;-2[\frac{\mu E(t)}{\hbar }-\beta S_{1}(t)]S_{2}(t)-\frac{S_{3}(t)+1}{T_{1}}\;.\; \end{array}$$

Here, *S*_1_(*t*) and *S*_2_(*t*) are, respectively, the mean real and imaginary parts of polarization, and *S*_3_(*t*) is the mean population inversion per electron (difference of the occupation probabilities in the upper and lower subbands). Also, *μ *=* ez*_01 _is the electric dipole matrix element between the two subbands, and the parameters *ω*_10_,*β*, and *γ* are given by

(4) $$\begin{array}{ll} \omega_{10}\; & =\;\frac{E_{1}-E_{0}}{\hbar }+\frac{\Pi e^{2}}{\hbar \varepsilon }N\frac{L_{1111}-L_{0000}}{2}\;,\; \end{array}$$

(5) $$\begin{array}{ll} \gamma \; & =\;\frac{\Pi e^{2}}{\hbar \varepsilon }N\left(L_{1001}-\frac{L_{1111}+L_{0000}}{2}\right)\;,\; \end{array}$$

(6) $$\begin{array}{ll} \beta \; & =\;\frac{\Pi e^{2}}{\hbar \varepsilon }NL_{1100}\;.\; \end{array}$$

Here, *N* is the electron sheet density, *ε *is the relative dielectric constant, *e* is the electron charge, *E*_0_ and *E*_1 _are the eigenvalues of energy for the ground and excited states in the well, respectively, and *L*_*ijkl *_= ∫∫* dzdz*^*′*^*ξ*_*i*_(*z*)*ξ*_*j*_(*z*^*′*^)|*z*−*z*^*′*^|*ξ*_*k*_(*z*^*′*^)*ξ*_*l*_(*z*), with *i*,*j*,*k*,*l *= 0,1. Also, *ξ*_*i*_(*z*) is the envelope wavefunction for the *i*th subband along the growth direction (*z*-axis). Finally, in Equations 1 to 3, the terms containing the population decay time *T*_1_ and the dephasing time *T*_2 _describe relaxation processes in the quantum well and have been added phenomenologically in the effective nonlinear Bloch equations. If there is no relaxation in the system *T*_1_,*T*_2_→*∞*, then $S_{1}^{2}(t)+S_{2}^{2}(t)+S_{3}^{2}(t)=1$.

In comparison with the atomic (regular) optical Bloch equations [49], we note that in the effective nonlinear Bloch equations, the electron-electron interactions renormalize the transition frequency by a time-independent term (see Equation 4). The parameter *γ*consists of two compensating terms: the self-energy term and the vertex term [20]. In addition, the applied field contribution is screened by the induced polarization term with coefficient *β*. The screening is due to exchange correction. Surprisingly, the exchange corrections appear with terms which are linearly dependent on the electron sheet density, as all exchange terms which present a nonlinear dependence on the electron sheet density are exactly canceled out due to the interplay of self-energy and vertex corrections [20].

For very short electromagnetic pulses, pulses that include only a few cycles, the field envelope may change significantly within a single period. In such a case, one should first define the vector potential and then use it to obtain the electric field; otherwise, unphysical results may be obtained [39-43,47,48]. So, the electric field *E*(*t*) is defined via the vector potential *A*(*t*) as *E*(*t*) = −*∂A*/*∂t*[39-43,47,48] where

(7) $$A(t)=A_{0}f(t)\cos(\omega t+\varphi )\;.$$

Here, *A*_0_ is the peak amplitude of the vector potential, *f*(*t*) is the dimensionless field envelope, *ω *is the angular frequency, and *φ*is the carrier envelope phase of the field. The form of the electric field becomes

(8) $$E(t)=\omega A_{0}f(t)\sin(\mathrm{\omega t}+\varphi )-A_{0}\frac{\mathrm{\partial f}}{\mathrm{\partial t}}\cos(\mathrm{\omega t}+\varphi )\;.$$

In the above formula, the first term corresponds to an electromagnetic pulse with a sine-oscillating carrier field, while the second term arises because of the finite pulse duration. This second term can be neglected for pulses with a duration of several cycles, but has an important effect in the single-cycle regime [39-43,47,48].

If the electron-electron interactions are neglected, then the nonlinear effective Bloch equations coincide with the optical Bloch equations of a two-level atom [49]. In this case, in the limit of no relaxation processes (*T*_1_*T*_2_→*∞*), if the ultrashort pulse effects are neglected and under the rotating wave approximation, the population inversion, with the initial population in the lower state, is given by

(9) $$\;S_{3}(t)=-\cos[\Lambda (t)],\Lambda (t)=-\overset{t}{\underset{0}{\int }}\frac{\mu \omega A_{0}f(t^{\prime })}{\hbar }dt^{\prime }\;,$$

where *Λ*(*t*) is the time-dependent pulse area [49]. At the end of the pulse, *Λ*(*t*) takes a constant value that is known as pulse area *θ*. Equation 9 clearly shows how important pulse area can be. If *θ *is an odd multiple of *Π*, then complete inversion between the two states is found at the end of the pulse, while if *θ* is an even multiple of *Π*, then the population returns to the lower state at the end of the pulse.

## Results and discussion

**Figure 1:**
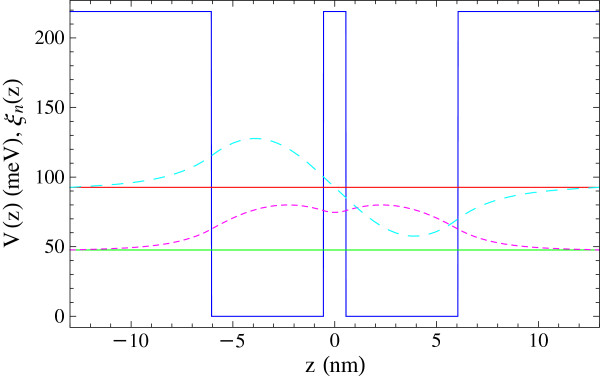
**Quantum well structure and corresponding envelope wavefunctions.** The confinement potential of the quantum well structure under study (blue solid curve) and the energies of the lower (green lower line) and upper states (red upper line). The envelope wavefunctions for the ground (dotted curve) and first excited (dashed curve) subbands.

In the current section, we present numerical results from the solution of the nonlinear Bloch equations, Equations 1 to 3, for a specific semiconductor quantum well system. We consider a GaAs/AlGaAs double quantum well. The structure consists of two GaAs symmetric square wells with a width of 5.5 nm and a height of 219 meV. The wells are separated by a AlGaAs barrier with a width of 1.1 nm. The form of the quantum well structure and the corresponding envelope wavefunctions are presented in Figure 1.

This system has been studied in several previous works [20,24,25,28,35-38]. The electron sheet density takes values between 10^9^and 7 × 10^11^cm^−2^. These values ensure that the system is initially in the lowest subband, so the initial conditions can be taken as *S*_1_(0) =* S*_2_(0) = 0 and *S*_3_(0) = −1. The relevant parameters are calculated to be *E*_1 _−* E*_0 _= 44.955 meV and *z*_01 _= −3.29 nm. Also, for electron sheet density *N *= 5 × 10^11^cm^−2^, we obtain *Πe*^2^*N*(*L*_1111_−*L*_0000_)/2*ε *= 1.03 meV, *ℏγ *= 0.2375 meV, and ℏβ=−3.9 meV. In all calculations, we include the population decay and dephasing rates with values *T*_1_= 10 ps and *T*_2_= 1 ps. Also, in all calculations, the angular frequency of the field is at exact resonance with the modified frequency *ω*_10_, i.e., *ω *=* ω*_10_.

**Figure 2:**
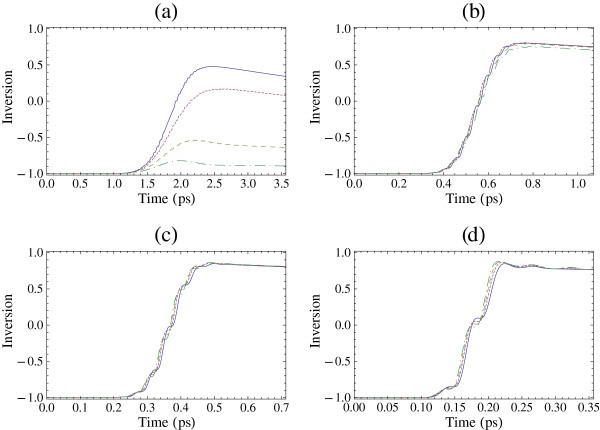
**The time evolution of the inversion *S***_**3**_**(*t*) for a Gaussian pulse.** The excitation is on-resonance, i.e., *ω *=* ω*_10_, the pulse area is *θ *=* Π*, and *φ*=0. (**a**) *n*_p_= 10, (**b**) *n*_p_= 3, (**c**) *n*_p_= 2, and (**d**) *n*_p_= 1. Solid curve: *N *= 10^9^cm^−2^, dotted curve: *N *= 3 × 10^11^cm^−2^, dashed curve: *N *= 5× 10^11^cm^−2^, and dot-dashed curve: *N *= 7 ×10^11^cm^−2^.

In Figure 2, we present the time evolution of the inversion *S*_3_(*t*) for different values of the electron sheet density for a Gaussian-shaped pulse with $f(t)=e^{-4\ln2{\left(t-2t_{p}\right)}^{2}/t_{p}^{2}}$. Here, *t*_p_= 2*Πn*_p_/*ω* is the duration (full width at half maximum) of the pulse, where *n*_p _is the number of cycles of the pulse and can be a noninteger number. The computation is in the time period [0,4*t*_p_] for pulse area *θ *=* Π*. For electron sheet density *N *= 10^9^cm^−2^, which is a small electron sheet density, Equations 1 to 3 are very well approximated by the atomic optical Bloch equations; therefore, a *Π *pulse leads to some inversion in the system in the case that the pulse contains several cycles. However, the inversion is not complete as the relaxation processes are included in the calculation and *T*_2_ is smaller than the pulse duration. In Figure 2a, that is for *n*_p_= 10, we see that the electron sheet densities have a very strong influence in the inversion dynamics. For example, for *N *= 3 × 10^11^cm^−2^, the population inversion evolves to a smaller value, and for larger values of electron sheet density, the final inversion decreases further and even becomes nonexistent.

A quite different behavior is found in Figure 2b,c,d for pulses with smaller number of cycles. In Figure 2b, we see that essentially the inversion dynamics differs slightly for *N *= 10^9^cm^−2^, *N *= 3 × 10^11^cm^−2^, and *N *= 5 × 10^11^cm^−2^ and all of these values lead to essentially the same final inversion. There is only a small difference in the inversion dynamics for the case of *N *= 7 × 10^11^cm^−2^ that leads to slightly smaller inversion. For even smaller number of cycles, Figure 2c,d, the inversion dynamics differs slightly for all the values of electron sheet density, and the final value of inversion is practically the same, independent of the value of electron sheet density. We note that the largest values of inversion are obtained for *n*_p _= 2 and *n*_p _= 3 and not for *n*_p _= 1, as one may expect, as in the latter case the influence of the decay mechanisms will be weaker. However, the second term on the right-hand side of the electric field of Equation 8 influences the dynamics for *n*_p _= 1, and in this case, the pulse area *θ *=* Π *does not lead to the largest inversion [42].

**Figure 3:**
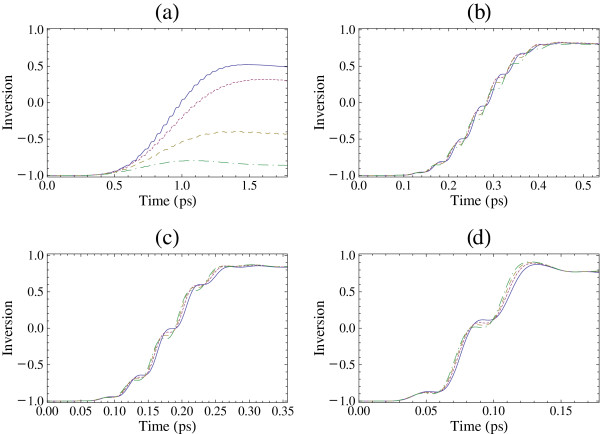
**The time evolution of the inversion *S***_**3**_**(*t*) for a sin-squared pulse.** The excitation is on-resonance, i.e., *ω *=* ω*_10_, the pulse area is *θ *=* Π*, and *φ*= 0. (**a**) *n*_p_= 10, (**b**) *n*_p_= 3, (**c**) *n*_p_= 2, and (**d**) *n*_p_= 1. Solid curve: *N *= 10^9^cm^−2^, dotted curve: *N *= 3 × 10^11^cm^−2^, dashed curve: *N *= 5 × 10^11^cm^−2^, and dot-dashed curve: *N *= 7 × 10^11^cm^−2^.

Similar results to that of Figure 2 are also obtained for the case of sin-squared pulse shape with $f(t)=\overset{2}{\sin}(\frac{\mathrm{\Pi t}}{2t_{p}})$ that are presented in Figure 3. In this case, the computation is in the time period [0,2*t*_p_] and the pulse area is again *θ *=* Π*. We have also found similar results for other pulse shapes, e.g., for hyperbolic secant pulses. These results show that the present findings do not depend on the actual pulse shape, as long as a typical smooth pulse shape is used.

**Figure 4:**
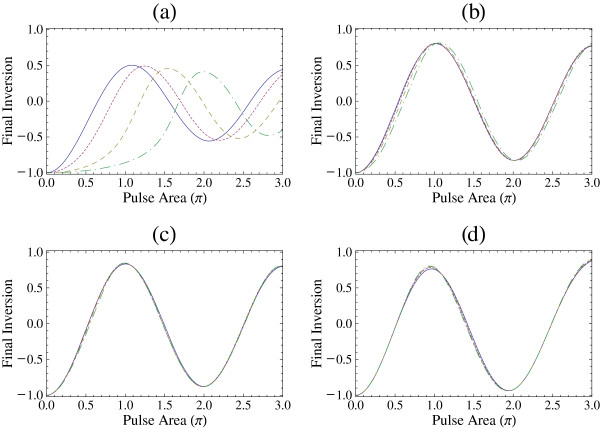
**Final inversion *S***_**3**_**(2*t***_**p**_**) for sin-squared pulse as a function of pulse area θ.** The pulse area is in multiples of *Π*. The excitation is on-resonance and *φ *= 0. (**a**) *n*_p_= 10, (**b**) *n*_p_= 3, (**c**) *n*_p_= 2, and (**d**) *n*_p_= 1. Solid curve: *N *= 10^9^cm^−2^, dotted curve: *N *= 3 × 10^11^cm^−2^, dashed curve: *N *= 5 × 10^11^cm^−2^, and dot-dashed curve: *N *= 7 × 10^11^cm^−2^.

In order to explore further the dependence of the inversion in pulse area, we present in Figure 4 the final inversion, i.e., the value of the inversion at the end of the pulse, as a function of the pulse area *θ *for a sin-squared pulse. We find that for pulses of several cycles, e.g., *n*_p _= 10, the pulse areas for maximum inversion can be quite different than *Π* depending on the value of electron sheet density. For example, for *N *= 5 × 10^11^cm^−2^, the pulse area is about 1.5*Π*, and for *N *= 7 × 10^11^cm^−2^, the pulse area is about 2.1*Π*. Similar results have also been obtained for other pulse shapes, e.g., Gaussian and hyperbolic secant pulses. The displayed dependence explains the results of Figure 3a (and of Figure 2a), as one may see that a *Π* pulse area leads to some final inversion for *N *= 10^9^cm^−2^ and *N *= 3 × 10^11^cm^−2^ but gives very small final inversion for *N *= 5 × 10^11^cm^−2^ and *N *= 7 × 10^11^cm^−2^. However, for pulses with 3 cycles or with a smaller number of cycles, the maximum inversion occurs for pulse area *Π* or very close to *Π* (and odd multiples of *Π *if the figures are extended in higher pulse areas) independent of the value of electron sheet density.

**Figure 5:**
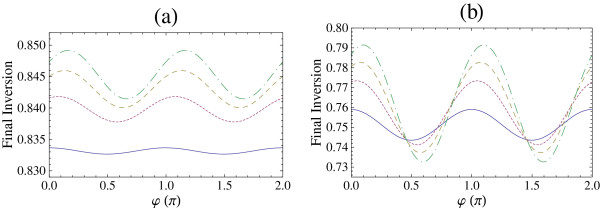
**Final inversion *S***_**3**_**(2*t***_**p**_**) for sin-squared pulse as a function of carrier envelope phase ***φ.* The carrier envelope phase is in multiples of *Π*. The excitation is on-resonance and the pulse area is *θ *=* Π*. (**a**) *n*_p_= 2 and (**b**) *n*_p_= 1. Solid curve: *N *= 10^9^cm^−2^, dotted curve: *N *= 3 × 10^11^cm^−2^, dashed curve: *N *= 5 × 10^11^cm^−2^, and dot-dashed curve: *N *= 7 × 10^11^cm^−2^.

An interesting effect in the interaction of an ultrashort electromagnetic pulse with a multi-level system is the influence of the carrier envelope phase *φ* on the populations of the quantum states [40,43,45,46,48]. In Figure 5, we present the dependence of the final inversion on the carrier envelope phase *φ* for a sin-squared pulse with *θ *=* Π *for different number of cycles and electron sheet densities. We find that there is a dependence of the final inversion on the carrier envelope phase and this dependence is strongest for larger sheet electron densities and for pulses with smaller number of cycles.

## Conclusions

In this work, we have studied the electron dynamics of intersubband transitions of a symmetric double quantum well, in the two-subband approximation, that is coupled by a strong pulsed electromagnetic field. We have used the effective nonlinear Bloch equations [20] for the description of the system dynamics, giving specific emphasis to the interaction of the quantum well structure with few-cycle pulses. We have found that high-efficiency population inversion is possible for specific pulse areas. The dependence of the efficiency of population transfer on the electron sheet density and the carrier envelope phase of the pulse has also been explored. More specifically, we have shown that for electromagnetic pulses with a duration of several cycles, the change in the electron sheet density leads to a very different response of the population in the two subbands to pulse area. However, electromagnetic pulses with pulse area *Π* or close to *Π* and with duration equal to or shorter than 3 cycles can lead to efficient population transfer between the two subbands independent of the value of electron sheet density.

## Competing interests

The authors declare that they have no competing interests.

## Authors’ contributions

EP conceived and developed the idea for the study, performed the simulations, and wrote the main part of the manuscript. JB contributed in the development of the original idea, in the analysis of the results, and in the writing of the manuscript. Both authors read and approved the final manuscript.

## Authors’ information

EP holds a PhD degree from the Physics Department of Imperial College from 1999. In 2001, he joined the Materials Science Department of the University of Patras, where he is currently an assistant professor. JB holds a PhD degree from the Department of Physics of the University of Patras from 1982. From 1991 to 2001, he was an assistant professor of Physics at the Department of Applied Sciences of the Technological Educational Institute of Chalkis. In 2001, he joined the Department of Renovation and Restoration of Buildings of the Technological Educational Institute of Patras, where he is currently a professor of Physics.
